# Approaches to embryonic neurodevelopment: from neural cell to neural tube formation through mathematical models

**DOI:** 10.1093/bib/bbae265

**Published:** 2024-06-08

**Authors:** Ali H Rafati, Sâmia Joca, Regina T Vontell, Gregers Wegener, Maryam Ardalan

**Affiliations:** Translational Neuropsychiatry Unit, Department of Clinical Medicine, Aarhus University, Palle Juul-Jensens Boulevard 11, 8200 Aarhus N, Denmark; Translational Neuropsychiatry Unit, Department of Clinical Medicine, Aarhus University, Palle Juul-Jensens Boulevard 11, 8200 Aarhus N, Denmark; Department of Biomedicine - Forskning og uddannelse, Vest, Aarhus University, Vest Ole Worms Allé 4 Bygning 1160, lokale 229, 8000 Aarhus C, Denmark; Department of Neurology, University of Miami Miller School of Medicine, Brain Endowment Bank, 1951 NW 7th Avenue, Suite 240 Miami, FL 33136, USA; Translational Neuropsychiatry Unit, Department of Clinical Medicine, Aarhus University, Palle Juul-Jensens Boulevard 11, 8200 Aarhus N, Denmark; Translational Neuropsychiatry Unit, Department of Clinical Medicine, Aarhus University, Palle Juul-Jensens Boulevard 11, 8200 Aarhus N, Denmark; Institute of Neuroscience and Physiology, Department of Physiology, Sahlgrenska Academy, University of Gothenburg, Medicinaregatan 11, 40530, Gothenburg, Sweden

**Keywords:** embryonic period, mathematical equations, neurodevelopment, natural transformation, topological spaces

## Abstract

The development of the human central nervous system initiates in the early embryonic period until long after delivery. It has been shown that several neurological and neuropsychiatric diseases originate from prenatal incidents. Mathematical models offer a direct way to understand neurodevelopmental processes better. Mathematical modelling of neurodevelopment during the embryonic period is challenging in terms of how to *‘Approach’,* how to initiate modelling and how to propose the appropriate equations that fit the underlying dynamics of neurodevelopment during the embryonic period while including the variety of elements that are built-in naturally during the process of neurodevelopment. It is imperative to answer where and how to start modelling; in other words, what is the appropriate ‘Approach’? Therefore, one objective of this study was to tackle the mathematical issue broadly from different aspects and approaches. The approaches were divided into three embryonic categories: *cell division, neural tube growth and neural plate growth.* We concluded that *the neural plate growth approach provides a suitable platform for simulation of brain formation/neurodevelopment compared to cell division and* neural tube growth. We devised a novel equation and designed algorithms that include geometrical and topological algorithms that could fit most of the necessary elements of the neurodevelopmental process during the embryonic period. Hence, the proposed equations and defined mathematical structure would be a platform to generate an artificial neural network that autonomously grows and develops.

## Graphical Abstract

**Graphical Abstract:**
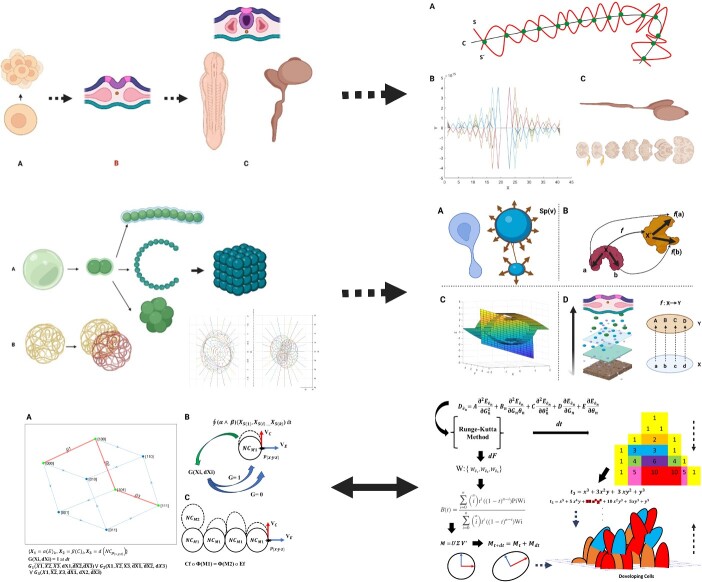

## Introduction

Human brain development is a process in which so-called neurodevelopment occurs according to a specific time frame. It starts in the third gestational week with differentiation of neural progenitor cells, lasting until late adolescence. Different types of cascades lead to neurodevelopment, from genetic mutations to environmental factors [1, 2]. However, there is no reliable parameter for early detection or prediction of alterations in central nervous system (CNS) development. Models that use mathematical concepts offer insight into the neurodevelopmental process and attempt to shed light on the underlying mechanisms of developmental processes in mathematical equations [3]. Another study discussed the theoretical and mathematical models in improving our understanding of neurodevelopment that would be helpful to fill the gap between system dynamics modelling in relation to subcellular processes [4]. Additionally, the field of cell migration and motility has benefited greatly from diverse mathematical modeling approaches, yielding significant contributions [5]. In mathematical modelling of neurodevelopmental processes, one of the biggest challenges is how to ‘approach’, meaning where to begin, and how to look at this entity for inspiration for computation/neural networks, but more importantly, to understand the underlying mechanisms that drive neurodevelopment during embryonic development [2, 6].

Our previous study addressed these questions at a preliminary level [7]. Eventually, we devised an equation that theoretically showed neuronal clustering in the cortex [8] by using cellular characteristics, but since such descriptions are limited [7], this study addresses these fundamental questions using more complex equations and algorithms. We explore the possibilities and pitfalls to understand how neurodevelopmental processes during embryogenesis can be analyzed mathematically. An embryonic neurodevelopmental dynamic modelling from this specific perspective is presented time in this study. Recent research [6] provided a mathematical model that frames how the brain network grows over the developmental period based on an equation that predicts the wiring between different brain regions. Using this framework, they showed diversity in neurodevelopment, a function of the wiring equation in terms of connectivity. However, details of the cellular components underlying neurodevelopment remain to be discovered and expressed [6]. Accordingly, our study emphasizes the importance of considering criteria such as continuity, complex-valued functions, and group homomorphism when mathematically modeling neurodevelopment by clarifying different types of mathematical modelling for neurodevelopmental processes as followed: Approach-1: Cell Division to Neurodevelopment; Approach-2: Neural Tube Development; Approach-3: Neural Plate Development. However, to provide a more precise definition of neurodevelopment in relation to embryonic period, we should mention that during embryonic period, the early stage is indicated by Morulae formation [9] which is defined by a mass of cells as a result of cell division, the next steps of embryonic period are mainly characterized by the natural process of neurulation which is indicated by proliferated cells in form of neural plate in which is transformed into neural tube [10, 11]. This study is based on biological observation with mathematical modelling. Hence, the significance of this study lies in its attempt to conceptualize, delineate, and classify neurobiological structures and electrochemical reactions into corresponding mathematical equations. This study necessitates a deeper mathematical exploration into neurobiological intricacies, such as the actin cytoskeleton and ion channels, which are integral components in neural network and quantum computation models. Understanding how the brain autonomously develops its circuits and neuronal structures, akin to mathematical automorphisms, could potentially bridge gaps in knowledge, aiding future endeavors to model neural networks and quantum computations more accurately.

## Methods

In this study, different mathematical equations and algorithms were generated. In case needed, the written equations in the form of functions were implemented in MATLAB (R2021B) to generate the plots. The latex of MATLAB was used for the shown formula. To make the data more comprehensible and be able to model neurodevelopment meaningfully according to what happens naturally, we divided the approach for modelling the neurodevelopment into following steps: 1′. Cell division, 2′. Neural tube Development, 3′. Neural Plate Development. They become comparable in mathematical terms based on the proposed models. Next, it is necessary to indicate the criteria that direct us to apply the appropriate mathematical entities and equations that are more compatible with biological structure: the first were holomorphic functions that indicate continuity and are defined by complex-valued functions; and also, to implicating group homomorphism, which is expressed by h: G→H. The group homomorphism implies that it can generate a preserved map while transforming from one structure to another one, which occurs for example during brain tissue growth and development. We applied rational Bézier curves (weighted Bernstein-form) to model the 3D shape of the actin network connecting to the cell membrane in Approach-1: from cell division to neurodevelopment. The following equations such as *Lambert W function*”, the Riemann–Siegel theta function [12], and gamma function have also been included to demonstrate a neuron unit schematically that could integrate mathematically into a tube like structure of CNS in Approach-2: from neural tube formation to neurodevelopment. We included the design of the bijective map with an automorphism that implies that the tissue in the plane regenerates and moves in the z-axis. Then, we defined the ‘*linear transformation’* [13] *and explained it in relation to the use of* ‘*Pascal’s triangle*’. Additional equations are the *Riemann–Siegel theta* function in conjunction with ‘*Weierstrass Sigma-Functio’* and ‘*elliptic function’* and ‘*partial differential equation’* (PDE) [14, 15] and the ‘*Runge–Kutta’* method [16, 17]. We addressed the singularity in our model by applying the Maclaurin series which are explained in detail in Approach-3: from neural plate formation to neurodevelopment. Further, we introduced how to model the neuronal morphology and development by regarding the electrical activity and chemical factors by using the topological spaces (such as *Lindelöf space, quotient space* [18], *Banach subspace* and *Hilbert space*) and applying the functors and natural transformation [19, 20]. In our model, we aimed to define neuronal growth and development through the integration of mathematical equations with the differentiation of a Boolean function, we applied the Gibbs free energy that combines the enthalpy and entropy. This approach allows us to determine the deterministic role of chemical reaction processes in guiding neuronal development along specific directions [21]. The Biorender (https://app.biorender.com/) was used to create the schematic illustrations.

## Results

### Approach-1: from cell division to neurodevelopment

**Figure 1:**
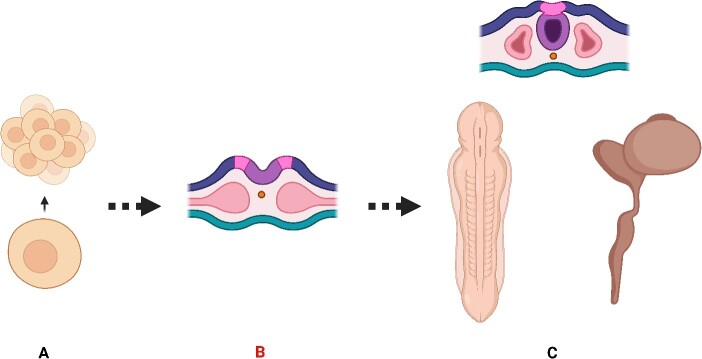
The schematic illustration of three steps of neurodevelopment. (A) Cell division where the pack of cells resemble ‘kissing number’ (B) Neural plate formation, (C) Neural tube formation.

**Figure 2:**
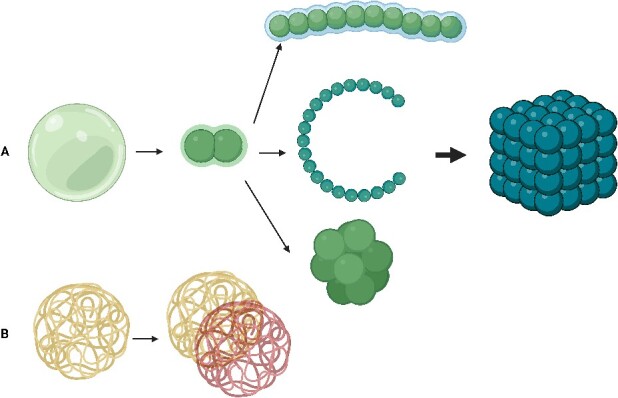
(A) Cell division polarization and the simple 3D cell organization. (B) The assumed cell-shell complex fibres called ‘cellular cortex’ correspond to the mathematical model shown in Fig. 3, it schematically shows how the division and polarity occurs in the dividing cell.

The stages of neurodevelopment is demonstrated in Fig. 1. Here, we explained how the cell–cell division and polarity play deterministic role in cells in relation to orientation and organization. In that case, if we consider cell division patterns and polarization as illustrated in Fig. 2A, we must be able to provide a mathematical model that fits all neurodevelopment steps. Still, it did not sound plausible based on the cell division and cellular polarization. We decided to use the cell membrane that is composed of complex filaments (Fig. 2B) which is called ‘cellular cortex’ for simulating the cell division. The ‘cellular cortex’ is an actin network connecting to the cell membrane containing myosin-2 motors. The function of the cellular cortex is to determine the cell shape, polarization, and actively participating in crucial cellular processes such as division, migration, and tissue morphogenesis [22, 23].

**Figure 3:**
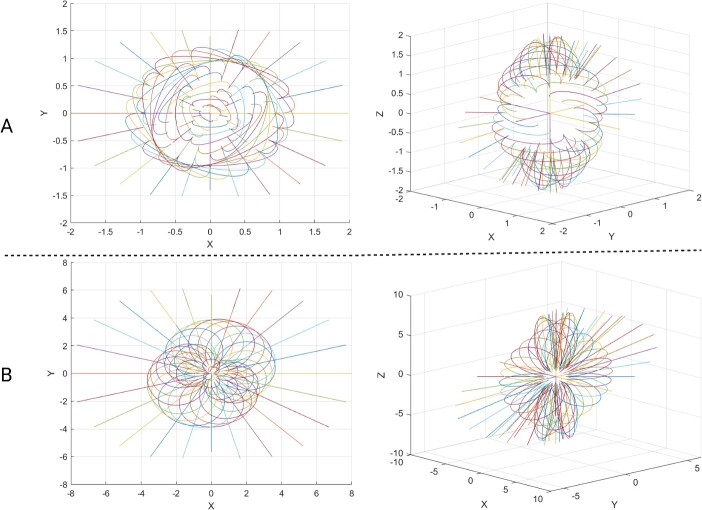
The applying complex values of “Wi'' to show the complex fibres. A the shapes of cells before polarization and division. (B) The shapes of cells after polarization and division. The radiated lines highlight how the curves are distributed in the space around the center of the dividing cell.

We applied the rational Bézier curves using the weighted Bernstein-form, the W stands for the weight that receives the complex values, and the Pi stands for the points and 0$\le t\le$ 1, which is shown in equation 1. We assumed that the generated convex body as shown in Figure 3A which indicates just before cell polarization and Fig. 3B which is just after cell polarization and division that are generated by using the sequential Bézier curves around the centre in 3D space simulate a cell that is dividing. Therefore for simplicity, we supposed to generate a sequence of curves to continue until to generate a tissue like structure shown in Fig. 2A, however, our model became limited to show only the dividing cells as shown in Fig. 2B. Equivalently, it is called ‘Winding Number’ which is defined by the number of times that a continuous curve travels around the certain point/points [24] (Fig. 3). Alternatively, one could think of the ‘kissing number’ [25] that is packing of spheres in a specific space, like in Fig. 1A.


(1) \begin{equation*} B(t)=\frac{\sum_{i=0}^n bi,n(t) Pi\ Wi}{\sum_{i=0}^n bi,n(t)\ Wi}\ \ or\ \ B(t)=\frac{\sum_{i=0}^n\left(\genfrac{}{}{0pt}{}{n}{i}\right){t}^i\ \left({\left(1-t\right)}^{n-i}\right) Pi\ Wi}{\sum_{i=0}^n\left(\genfrac{}{}{0pt}{}{n}{i}\right){t}^i\ \left({\left(1-t\right)}^{n-i}\right)\ Wi} \end{equation*}

However, it implies that we are restricted in modelling the biological structures mathematically since approximation to the biological structure by applying mathematical models is rather sophisticated. Consequently, the pitfalls and possibilities are remarkable for this approach, ‘From Cell Division to Neurodevelopment’. Therefore, as this approach did not help to continue modelling for the rest of the brain development, we used another equation by applying a different mathematical method instead. Creating a simulated tissue to illustrate the natural course of neurodevelopment proves challenging due to its complexity.

### Approach-2: from neural tube formation to neurodevelopment

**Figure 4:**
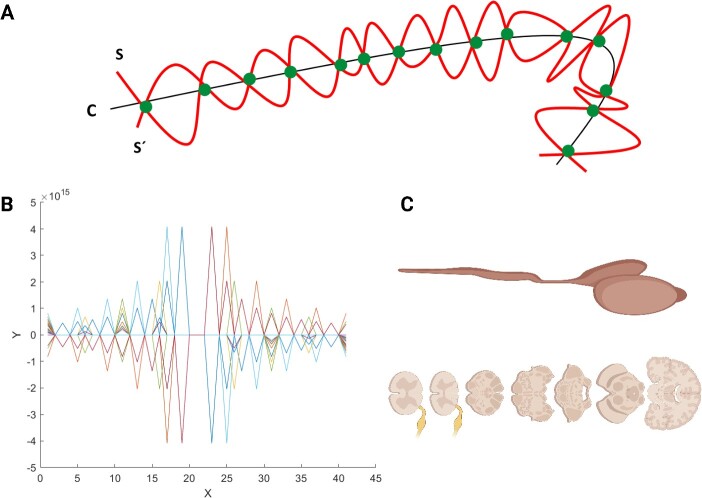
(A) Three curves C, S, S´. The S and S´ cross, the midline curve C, symmetrically looks like the CNS trajectories crossing all along, designed similar to ‘Möbius plane’. (B) The plot is the mathematical equivalence of the speculation in A, as it shows here, it is a symmetrical distribution of two curves around the midline curve. (C) Two schematic illustrations are representative of CNS including brain and spinal cord.

In this approach, we defined a neural tube development similar to the Möbius plane [26]. Here, we designed *B*: = {C, S, S´} in which C indicates the curve like equation 2 and S & S´ indicates two spaces (equivalent to two brain hemispheres and symmetry in CNS) that cross at crossing points on curve C as shown in Fig. 4A.


(2) \begin{equation*} B:= \left\{C,S,{S}^{\acute{\mkern6mu}}\right\},B(t)=\left(C(t),S(t),{S}^{\prime }(t)\right)\ \mathrm{in}\ {R}^2 \end{equation*}

In fact, equation ‘B (t)’ is helpful in guiding its appropriate parametrization as shown in equation 3 that is demonstrated in Fig. 4B. Thus, this equation reflects the symmetry that we observe in the CNS.


(3) \begin{equation*} L=\zeta \left({e}^{\pi xy1i}\right),-20\le x\le 20,-1\le y\le 1, \end{equation*}

*i* is an imaginary value.

**Figure 5:**
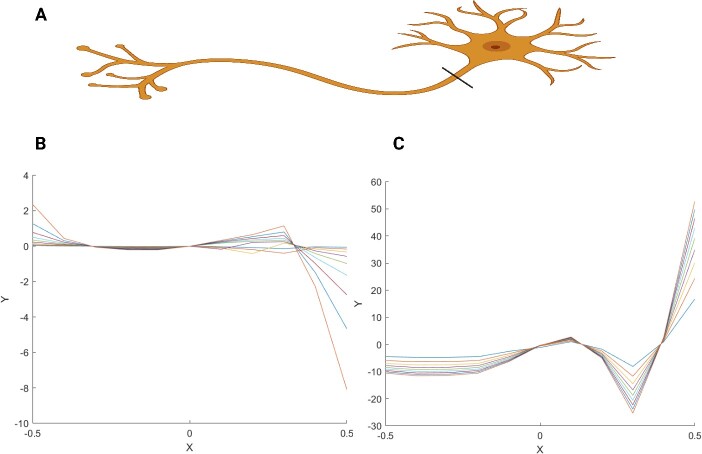
(A) It shows a neuron with its long axon. (B and C) They demonstrate two different equations with similar shapes that can reflect single-unit neurons. It is assumed that theoretically, using this unit would help the mathematical neurodevelopment model like Fig. 4C.

We further used equation 3 to generate a single-cell neuron model. Furthermore, we developed Equations 4 and 5 by applying the ‘*Lambert W function*’ and the Riemann–Siegel theta function [12], a gamma function that has also been included in both equations 3 and 4. Using these two equations we were able to demonstrate a neuron schematically (Fig. 5A). They demonstrate two different equations with similar shapes that can reflect single-unit neurons (Fig. 5B and C). This single neuron can be integrated into the mathematical model of CNS structure similar to the model in Equation 3; however it shows limited options when we try to mathematically design the model by inserting the single neuron as a unit building block for model ‘B(t)’ shown in Fig. 4. However, despite providing these equations for single neuron and CNS models at this step via this specific approach, it seems impossible to give a *general equation* covering all neurodevelopment stages. Accordingly, this is the downside of this model since we could not clearly define how cells could be built up through this model and via this approach in light of the complexity of the CNS and especially the trajectories. We decided to develop an equation that could deal with most of the issues of cell build-up and trajectories, if not.


(4) \begin{align*}& t=\frac{2\pi \left(n+\frac{1}{8}\right)}{w_0\left({e}^{-n-\frac{1}{8}}\right)},\nonumber\\&\theta (t)=-\frac{\ln \left(\pi\ t\right)}{2}-\frac{\ln \left(\varGamma \left(\frac{1}{4}+\frac{ty1i}{4}\right)\right)-\ln \left(\varGamma \left(\frac{1}{4}+\frac{ty1i}{4}\right)\right)1i}{2},\\&\mathrm{if}\ T=\theta (t),\mathrm{then}\ F={e}^{\pi\ T\ x\ y},-20\le x\le 20,0.1\le y\le 1,\nonumber\\&\mathrm{and}-2\le n\le 6.\nonumber \end{align*}

(5) \begin{align*} &\theta (t)=-\frac{\ln \left(\pi\ t\right)}{2}-\frac{\ln \left(\varGamma \left(\frac{1}{4}+\frac{ty1i}{4}\right)\right)-\ln \left(\varGamma \left(\frac{1}{4}+\frac{ty1i}{4}\right)\right)1i}{2},\\&\mathrm{if}\ T=\theta (t),\mathrm{then}\ F={e}^{\pi\ T\ x\ y},-20\le x\le 20,0.1\le y\le 1,\nonumber \end{align*}

and *t* is imaginary numbers.

### Approach-3: from neural plate formation to neurodevelopment

This approach requires exploring the factors determining the ultimate 3D structure of a tissue and organ such as the brain, heart, etc. The relevant mathematical theory, called ‘Representation Theory’, explores how linear transformation is applied to vector spaces and geometrical objects and studies topological methods.

**Figure 6:**
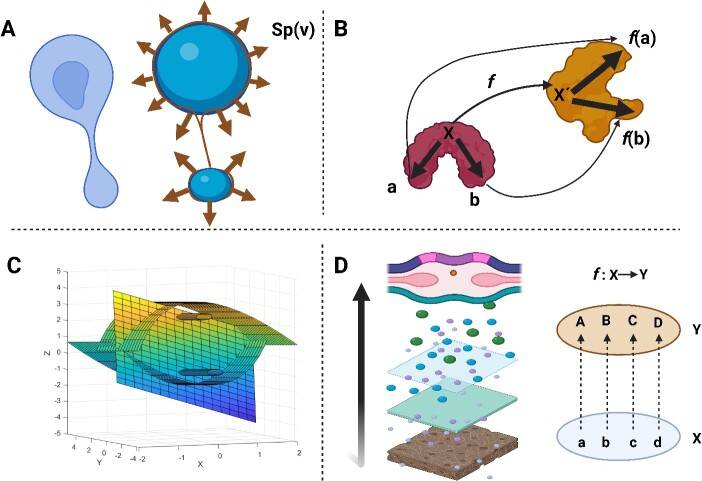
(A) The left object shows a shape change in a spheroid cell, while the right shows two blue spheres reflecting the shape operator (${S}_p(v)$=$-{\nabla}_vN$) with unit normal vectors (N) on the brown curves that show how the curves are moving in every direction during the cellular shape change. (B) The mapping and transformation are illustrated clearly by an example of how transformation might occur using (f: X → x´). (C) An example of a so-called parabolic cylinder coordinate shows the spatial orientation and organization of cylinders in relation to manifolds. D. It shows how the cells migrate and organize through the layers from bottom to top, like in a neural plate. The last item shows the bijective function (*f*: X → Y), an isomorphism, and its map is an example of mathematical modelling of tissue growth.

We postulated that to generate a 3D structure of CNS geometrically, there should be a 3D map along the z-axis to guide us on how to grow the tissue in a stepwise manner according to a certain numerical map (Fig. 7.2). Therefore, we need to design the bijective map (Fig. 6D) similar to Cayley Groups table [26] such that F: ${G}_1\to{G}_2$, however it differs in our model based on how the changes occur on the table. As there is an automorphism in our model such that the tissue in the (x-y) plane regenerates itself, the mapping and changes on the (x–y) plane are transferred in the z-axis direction according to Fig. 7.2A and B. This process is equivalent to neuronal tissue growth. To determine the amount of cell growth and development, we need a numerical map that is geometrically isomorphic [27] on the z-axis from one layer of tissue to another.

**Figure 7.1:**
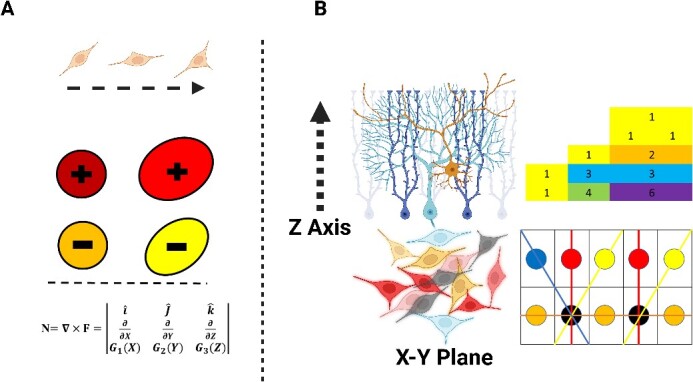
(A) Schematic illustration shows how the neuronal progenitors change shape and direction during neurodevelopment. This illustration is related to our previous study [7], in which we designed how cell shape, direction, and electrical activity are defined as independent elements for neurodevelopment. The 3D position of cells in beginning = {$\hat{i}$, $\hat{J}$, $\hat{k}$}, the G function and its parameters as the change in position of cells = {d${p}_1$, d${p}_2$, d${p}_3$}, shapes = sphere, ellipsoid, pyramidal approximation, other convex bodies, electrical activity = excitatory {$+$}, inhibitory {$-$}, excitatory/inhibitory {$\pm$}, X ($d{p}_1$, s, ${e}_1$), Y ($d{p}_2$, s, ${e}_2$), Z ($d{p}_3$, s, ${e}_3$). (B) It represents the ‘*linear subspace’* in a finite field indicated by circles and connecting linear. The Pascal’s triangle is located above it. The schematic illustration of colored cells in the x-y plane is analogous to *linear subspace”* in a finite field. The neuronal growth and organization in the z-axis are shown.

As a result, we provide some examples of a (x–y) plane and a numerical map demonstrated in Fig. 7.1A and B, respectively. Figure 7.1B shows that the numerical map could be a Pascal triangle. Thus, the z-axis must be assigned to a numerical map for each tissue/organ individually, which might be considered specific and constant for that tissue/organ. Similarly, the analogous numerical map to the Pascal triangle is the well-known Hermite polynomials [28], ${H}_n(x)={\left(-1\right)}^n{e}^{x^2}\frac{d^n}{d{x}^n}\ {e}^{-{x}^2}$ which yields the following polynomials of degree n: ${\mathrm{H}}_0\left(\mathrm{x}\right)$=1; ${\mathrm{H}}_1\left(\mathrm{x}\right)$= $2\ \mathrm{x}$; ${\mathrm{H}}_2\left(\mathrm{x}\right)$= 4${\mathrm{x}}^2-2$; ${\mathrm{H}}_3\left(\mathrm{x}\right)$= 8${\mathrm{x}}^3-12\ \mathrm{x}$.

Therefore, before explaining our equation for this approach, we provide some relevant mathematical equations that could be equivalent to our equation, summarized in Fig. 7.3. They must provide information about the curves, surface, or transformation from one shape to another. However, to meet our requirements for mathematically designing a tissue/organ. They are collectively summarized in Fig. 6. The first function of this kind is called the ‘*shape operator*’ [29–31], which is defined by: ${S}_p(v)$=$-{\nabla}_vN$. The *p* stands for a point on surface *M*, the normal vector at point *p* is *N,* and *v* is a tangent vector on *M* at *p.* Then, any change in the surface area around the point *p* can be estimated. It has been demonstrated in Fig. 6A. It provides information about the shape of curves in terms of their normal vector on the curves and the direction of change in curvatures. Another definition related to the shape operator uses principal curvatures and principal directions, equivalently called ‘*eigenvalues and eigenvectors’*. Thus, the parameters define the shape change of a geometrical object (Fig. 6A).

Moreover, the relevance to shape transformation could be obtained by applying the ‘*linear transformation’* [13]. Generally, the ‘*linear transformation’,* which is also called ‘*linear mapping* or *linear operator’,* is defined as follows: if X Y considered vector space, then there would exist x and y as vectors that belong to X and Y, respectively and y is the image of x by *F* function, expressed by y = F(x), (Fig. 6B).

Another relevant concept involves dynamical systems associated with differential geometry, which examines the configuration of manifolds. Dynamical systems can be classified into nonlinear and linear categories [32]. As an example, the ‘*Koopman operator’* [33] is defined as an infinite-dimensional operator that applies to nonlinear dynamic systems. Another example is orthogonal polynomial indicated by orthogonal functions in 3D space.

Another related entity applicable in the geometrical application to dynamic systems is the ‘*parabolic cylinder coordinate’* [34]. That can be expressed based on a hypergeometric function. As the cylinders organize and orient concerning manifolds, this mathematical analogy can be applied to organizing tissue/organ geometrically (Fig. 6C). The parabolic cylinder function is denoted by ${D}_v(z)=$  ${2}^{\left({}^{v}\left/ _{2}\right.\right)}{e}^{\left({}^{-{z}^2}\left/ _{4}\right.\right)}U\left(^{-1}\left/ _{2}\right.v,^{1}\left/_{2}\right.,^{1}\left/ _{2}\right.{z}^2\right)$ [35].

In the next step, two essential steps of our main equation are presented in simplified form by mathematical equations explained in Figure 7. At this step, we provide more information about our equation by defining the ‘*linear/vector subspace’*.

If we assign the ‘*linear/vector subspace’* as A in a finite field [36] of B, which is R^2^, then we can obtain the linear equations related to the subspace by setting the points (x, y) in R^2^. If fixed vectors are added to a vector/linear subspace, it could be d ‘*affine space’* [37]. Thus, the affine subspace is denoted by having a subspace B that belongs to affine space A such that by setting the points b$\in B$ and containing vectors $\overrightarrow{C}$ that make the linear subspace B. Hence, for every point such that ${b}_n\in B$, there is a vector called $\overrightarrow{C}$ that projects the point ${b}_n$ by function F. In general, the subspacebelongs to modular lattices (Dedekind lattices) which has been defined elsewhere [38].

**Figure 7.2:**
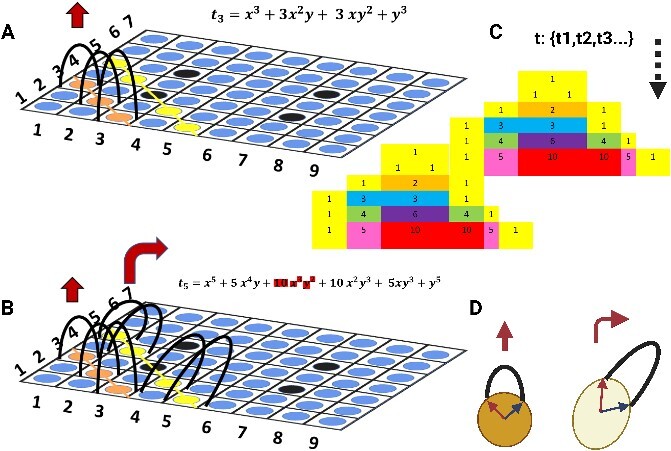
(A) The schematic illustration shows the steps that cells grow vertically using a ‘*linear subspace*’, as explained in the Fig. 7.1, then we have shown the vertical ‘black curves’, which are indeed ‘*Bezier curve*’ generated by using equation 1 and grows vertically/obliquely according to the *Pascal triangle*, for example, the ${t}_3$ generated by the 3^rd^ line of the Pascal triangle and is along the orange circles while connecting the ‘3’ points; they are curvilinear lines that determine the whole shape of tissue in that certain section. However, the height and growth direction of each cell are more sophisticated and determined by curves that are a function of ‘W’ calculated from equation 1 and ‘M’ (see text), respectively. The circles represent different cell types with their specific properties, summarized in ‘M’. (B) It shows the next steps of the growing that there is a ‘*yellow line.’*  ${t}_5$ through the circles generated based on the ‘*specific row*’ in the ‘*Pascals triangle*’. As shown, the black circle could be a ‘*singularity’*, so it differs from other circles therefore that one can be removed $\mathbf{10}\ {\boldsymbol{x}}^{\mathbf{3}}{\boldsymbol{y}}^{\mathbf{2}}$ (see text) and the rest of the equation will appear differently when plotted. This singularity can be equivalent to a specific cell type or extracellular matrix. (C) It shows two consecutive limited ‘*pascals triangle*’; the binominal coefficient yields it and generates the equations (t_1_:t_n_) accordingly. (D) It depicts a circle and an ellipsoid; they have two curves generated by two vectors using equation 1, ‘*Bezier curve*’.

**Figure 7.3:**
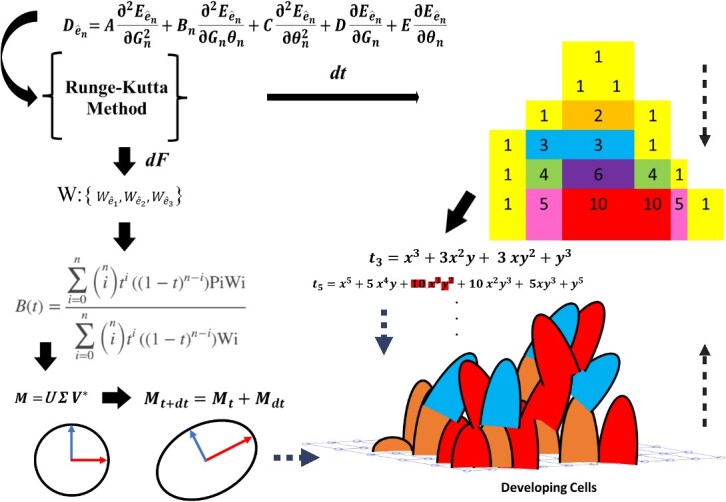
The schematic illustration of the steps of the whole mathematical process of our proposed model. It starts with the equation, ends in developing cells and repeats continuously until complete implementing according to, as an example, *the Pascals triangle*.

Furthermore, we generated the linear/vector subspace in relation to polynomial expansion using ‘*Pascal’s triangle’* [39] as a binomial coefficient (Figure 7.2C). The summarized explanation of the equation is presented in Fig. 7.2. This explanation needs to address why we employ ‘*Pascal’s triangle’* and its connection to polynomial expansion. Each 3D tissue/organ formation must be assigned a set of magic numbers that guides how much the tissue/organ must develop numerically in the z-axis to achieve the final 3D shape. These magic numbers may vary depending on the specific tissue/organ of the animal/human. Consequently, we proposed the equation in Fig. 7.3 which elucidates the relationship between the ‘*linear/vector subspace’* and ‘*Pascal’s triangle’,* which is serving as a set of magic numbers for neuronal tissue formation. Nevertheless, another study is necessary to explore how these individualized magic numbers are assigned to different tissues and organs.

In the next step, we defined our main equation that is obtained accordingly, as shown below in equation 6. We first defined the elements and main contributors to the equation 6, including the formulated cellular properties in conjunction with the *Riemann–Siegel theta* function shown in equation 5. The similar operations regarding our introduced equation 6 have been demonstrated by discussing the ‘*Weierstrass Sigma-Functio’* and ‘*elliptic function’* and ‘*partial differential equation’* (PDE) [14, 15]. The following steps demonstrated how the partial differential equation (PDE) is applied to generate the equation 6 by using the ${\mathbf{G}}_{\boldsymbol{n}}$, $\boldsymbol{\theta}\left({\boldsymbol{G}}_{\boldsymbol{n}},\boldsymbol{t}\right)$ and $\boldsymbol{E}\left({\boldsymbol{G}}_{\boldsymbol{n}},{\boldsymbol{\theta}}_{\boldsymbol{n}}\right)$**,** sequentially. It implies how the cellular properties acts through the fowling mentioned equations to affect the cell cycle and growth in the schematic illustration.

▪ ${\mathbf{G}}_{\boldsymbol{n}}$= $\left[{\mathrm{G}}_1\left(\mathrm{X}\right),{\mathrm{G}}_2\left(\mathrm{Y}\right),{\mathrm{G}}_3\left(\mathrm{Z}\right)\right]$, ${G}^{\prime }$= *N,*  ${x}_i=\left[X,Y,Z\right]\ \mathrm{and}\ {x}_i\in R$
▪ ***N***$$=\nabla \times F=\left|\begin{array}{ccc}\hat{i}& \hat{J}& \hat{k}\\{}\frac{\partial }{\partial X}& \frac{\partial }{\partial Y}& \frac{\partial }{\partial Z}\\{}{G}_1(X)& {G}_2(Y)& {G}_3(Z)\end{array}\right|$$*, the cellular properties*

\begin{align*}\kern-.8pc \boldsymbol{N}=&\left[\left(\frac{\partial{G}_Z}{\partial Y}-\frac{\partial{G}_Y}{\partial Z}\right)i+\left(\frac{\partial{G}_X}{\partial Z}-\frac{\partial{G}_Z}{\partial X}\right)j+\left(\frac{\partial{G}_Y}{\partial X}-\frac{\partial{G}_X}{\partial Y}\right)K\right]\approx\\& \left[\begin{array}{c}\left(\frac{\partial{G}_Z}{\partial Y}-\frac{\partial{G}_Y}{\partial Z}\right)i\\{}\left(\frac{\partial{G}_X}{\partial Z}-\frac{\partial{G}_Z}{\partial X}\right)j\\{}\left(\frac{\partial{G}_Y}{\partial X}-\frac{\partial{G}_X}{\partial Y}\right)K\end{array}\right] \end{align*}

\begin{align*}&\kern-1pc \boldsymbol{\theta}\left({\boldsymbol{G}}_{\boldsymbol{n},}\boldsymbol{t}\right)=\\&\frac{-\ln \left(\pi\ t\right)}{2}-\frac{\ln \left(\varGamma \left(\frac{1}{4}+\frac{t\ {G}_n\ 1i}{4}\right)-\ln \left(\varGamma \left(\frac{1}{4}-\frac{t\ {G}_n\ 1i}{4}\right)\right)\right)1i}{2},\\&n\in \left\{1,2,3\right\} \end{align*}

▪ $\boldsymbol{E}\left({\boldsymbol{G}}_{\boldsymbol{n}},{\boldsymbol{\theta}}_{\boldsymbol{n}}\right)= \left({e}^{G(X)\pi {\theta}_1}\right){\hat{\boldsymbol{e}}}_{\mathbf{1}}+\left({e}^{G(Y)\pi{\theta}_2}\right){\hat{\boldsymbol{e}}}_{\mathbf{2}}+\left({e}^{G(Z)\ \pi {\theta}_3}\right){\hat{\boldsymbol{e}}}_{\mathbf{3}}$
▪ $\boldsymbol{Ah}\left(\boldsymbol{x}\right)=H\left(x,h(x),\dots, {\partial}_{x1\dots xn}^mh(x)\ \right)$, differentiable function of *F,* then the 2nd order partial differential equation (PDE) is defined as **D**$\left(\boldsymbol{x}\right)=\sum_{1\le j\le k\le N}{C}_{j,k}(x){\partial}_{j,k}^2h(x)+\sum_1^N{C}_i(x){\partial}_ih(x)$ [40, 41].

$$ n\in \left\{1,2,3\right\},A=C=D=E=1; $$

$$ {B}_i=\left(\frac{\partial{G}_Z}{\partial Y}-\frac{\partial{G}_Y}{\partial Z}\right)i,{B}_j=\left(\frac{\partial{G}_Z}{\partial Y}-\frac{\partial{G}_Y}{\partial Z}\right)j,{B}_K=\left(\frac{\partial{G}_Y}{\partial X}-\frac{\partial{G}_X}{\partial Y}\right)K $$

(6) \begin{equation*} \kern0.50em {\boldsymbol{D}}_{{\hat{\boldsymbol{e}}}_{\boldsymbol{n}}}=A\frac{\partial^2{E}_{{\hat{\boldsymbol{e}}}_{\boldsymbol{n}}}}{\partial{G}_n^2}+{B}_n\frac{\partial^2{E}_{{\hat{\boldsymbol{e}}}_{\boldsymbol{n}}}}{\partial{G}_n{\theta}_n}+C\frac{\partial^2{E}_{{\hat{\boldsymbol{e}}}_{\boldsymbol{n}}}}{\partial{\theta}_n^2}+D\frac{{\partial E}_{{\hat{\boldsymbol{e}}}_{\boldsymbol{n}}}}{\partial{G}_n}+E\frac{\partial{E}_{{\hat{\boldsymbol{e}}}_{\boldsymbol{n}}}}{\partial{\theta}_n} \end{equation*}

We showed the black curves that stretch vertically; they are the ‘*Bezier curve*’ that their vectors of ‘M’ generate them, and the curve is controllable by ‘W’, so ${M}_{t+ dt}={M}_t+{M}_{dt}$, the ‘t’ is obtained by the ‘*Runge–Kutta’* method [16, 17]. The ‘*Runge–Kutta’* method is “${K}_1= hf\left({x}_n+{y}_n\right)$; ${K}_2= hf\left({x}_n+\left(1/2\right)h,{y}_n+\left(1/2\right){k}_1\right)$; ${y}_{n+1}={y}_n+{K}_2+$ O (${h}^3$)”, which is a number solution method to our equation 6 and generates input${D}_{{\hat{e}}_n}$={${D}_{{\hat{e}}_1},{D}_{{\hat{e}}_2},{D}_{{\hat{e}}_3}$} for W = {${W}_{{\hat{e}}_1},{W}_{{\hat{e}}_2},{W}_{{\hat{e}}_3}$} in three axes. Therefore, the ‘*Bezier curves’* are generated in three axes like a 3D object which is not shown here and only one direction is shown in Fig. 7.3. In addition, the singular value decomposition is denoted by $M=U\sum{V}^{\ast }$ in which U and V unitary matrices and $\varSigma$ is a diagonal matrix.

In the next step, we explained the ‘B(t)’ in Figure 7.3 is a so-called *binomial formula* that generates the *Pascals triangle;* meanwhile, it uses${t}_{\mathbf{1}:\boldsymbol{n}}$to assign the equation of ${t}_3$ and$\kern0.50em {t}_5$ for developing cells (linear subspace), as an example, is shown in Figure 7.3*.* When these curvilinear lines are generated by *a* binomial formula, they determine the whole shape of developing tissue structure in that section. If we consider ${t}_3$  $and\kern0.50em {t}_5$, this expression $\mathbf{10}\ {\boldsymbol{x}}^{\mathbf{3}}{\boldsymbol{y}}^{\mathbf{2}}$ from ${t}_5$ could be removed as this third part is equivalent to the singularity point in Fig. 7.2B. Regarding the singularity, the Maclaurin series can be applied based on the singularity, non-isolated set such as a boundary with no analytic function around it [42], but in case of isolated singularity point can be explored by Laurent expansion, $f(z)=\sum_{-\infty}^{\infty }{a}_n{\left(z-c\right)}^n$, ${a}_n$ is the coefficient and also in case of meromorphic function (complex function), can be used and investigated for singularities.

Next in the below, ‘c’ shows the ${t}_5$ transforms to ${t}_{5,2}$ Then, the new plot is generated from the new equation to generate a different plot depending on the equations. We think this also happens in biology when the type of the cells varies along a certain cell line, so in this case, they can be considered as singularity point/points. That point is removed from the equation whenever the singularity *point*/*points* are not replaceable by Laurent expansion.

a) $B=\sum_{k=0}^n\left(\genfrac{}{}{0pt}{}{n}{k}\right)\ {x}^{n-k}{y}^k$, $if\ k\le n\&{t}_{\mathbf{1}:\boldsymbol{n}}$

$$ {t}_3={x}^3+3{x}^2y+3\ {xy}^2+{y}^3; $$

b) ${t}_5={x}^5+5\ {x}^4y+\mathbf{10}\ {\boldsymbol{x}}^{\mathbf{3}}{\boldsymbol{y}}^{\mathbf{2}}+10\ {x}^2{y}^3+5x{y}^3+{y}^5$ → ${t}_{5,2}={x}^5+5\ {x}^4y+10\ {x}^2{y}^3+5x{y}^3+{y}^5$
c) $\left\langle 1\ 2\ \mathbf{3}\boldsymbol{singularity}\ 4\ 5\ 6\ \right\rangle$It is the third cell on the orange line that is singularity, so we remove the 3rd on the equation.

Briefly, the whole process is summarized in Figure 7.3. Further, we provide an example for 3D modelling of neuronal tissue formation and development according to Figure 7.3 which is shown in Fig. 9.

**Figure. 8:**
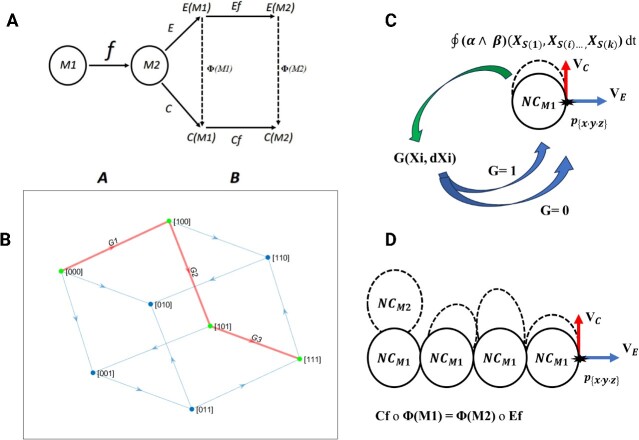
The summary of the equation involved in NC growth and development. (A) It demonstrates the diagram of the Functors and the natural transformation denoted by Φ and how commutes: Cf o Φ(M1) = Φ(M2) o Ef. (B) It demonstrates an example of a differential Boolean function in the form of graph G (xi, dXi) and shows how dXi changes are dependent on xi changes. The xi represents the factors ${X}_1=\alpha{(E)}_k$, ${X}_2=\beta{(C)}_l,{X}_3=d\left({NC}_{{\boldsymbol{p}}_{\left\{x,y,z\right\}}}\right)$ that determine how the dXi changes, for example, ($\overline{X1},\overline{X2},\overline{X3}$) = [0,0,0] can change in value as follows (X1$, \overline{X2},\overline{X3}$) = [1,0,0]. The G1($\overline{X1},\overline{X2},\overline{X3}$, dX1$\overline{dX2},\overline{dX3}$) ⋁ G2($\mathrm{X}1,\overline{X2},\overline{X3}$, $\overline{dX1}$,$\overline{dX2},\mathrm{dX}3$) ⋁ G3($\mathrm{X}1,\overline{X2},\mathrm{X}3$, $\overline{dX1}$,$\mathrm{dX}2,\overline{dX3}$) are shown in red with distinguished pathways accordingly. (C) It shows the relation between the factors involved in cell growth and development for every point of p of manifold, NC(M1). It needs to implement the interaction of vector fields on NC(M1) such that $\oint \left(\alpha \wedge \beta \right)\left({X}_{S(1)},{X}_{S(i)\dots, }{X}_{S(k)}\right) dt$, it integrates each time (dt) that receives the g = 1 in which the g is a function of xi changes in the form of inputs. (D) The whole process of NC(M1)→NC(M2) is shown here. The vector fields of VC and VE at point P{x, y, z} are shown. The Cf o Φ(M1) = Φ(M2) o Ef represents the interaction of functors and vector fields, and natural transformation is reflected as the schematic illustration in the form of interaction between NC and vector fields.

Alternatively, we can model the neuronal cell morphology and development in connection with electrical activity and chemical factors to explore how it naturally would change and evolve. Therefore, concerning topology, we can apply the functors and natural transformations [19, 20] from category theory. These functions are defined by the morphism and objects as demonstrated in Fig. 8. Each category has its own certain structure, with functions consisting of elements known as objects belonging to a certain category. The morphisms are canonical maps, for example**,**  $\boldsymbol{f}:\mathrm{M}1\longrightarrow \mathrm{M}2$, the M1 and M2 are denoted as objects, and *f* is the morphism that maps from M1 to M2. The functors of our diagram are defined as a parallel pair E and C, from categories A and B such that $E,C:\boldsymbol{A}\boldsymbol{\Rightarrow}\boldsymbol{B}$.

**Figure 9:**
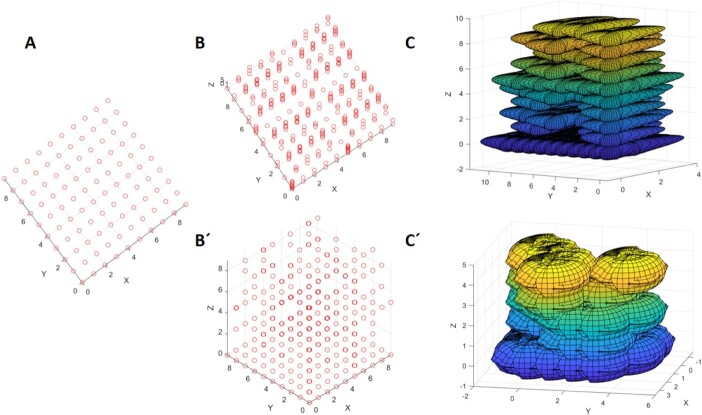
It demonstrates an example of using a numerical map plus neuronal properties to generate a mathematical model of neurodevelopment in a 3D tissue-like structure as shown in error! Reference source not found 7.3. (A) It shows a lattice distribution of points related to elliptic curve P(x, y) = ${y}^2-{x}^3- ax-b=0$ on a finite field, if the remnant of P(x, y) which is r (x, y) = 0, then we can generate all the points on the curve in plane. (B) Next, we can generate a homogenous polynomial which is P(x, y, z) = ${zy}^2-{x}^3- ax{z}^2-{bz}^3=0$, (b = 0), to generate a 3D point distribution organized vertically like neuronal organization shown in (view B and view B´). (C) Next, the two plots shows the distribution of pyramidal (C) and non-pyramidal cells (C´) respectively, generated accordingly by equations that are introduced in our previous study [7] and contains the neuronal shape properties including the nucleolus in the center and these nucleolus are located on all of these points. Therefore, the deterministic factor that indicates the next location of the neurons is dependent on these points that are located on elliptic curve with the mentioned condition that function as a numerical map.

In Fig. 8, the first category is called ‘*A*’ that contains $f:\mathrm{M}1\longrightarrow \mathrm{M}2$, indicating that M1 and M2 are objects, and M2 is mapped by morphism ‘*f’*. The M1 and M2 represent manifolds of a certain type of neuronal cell that. In the next step within category ‘*B*’, is transformed into E(M1) and C(M1), representing electrical activity which is Functor ‘E’ and chemical factors which is Functor ‘C’, respectively. They are involved in influencing the cell morphology (M: manifolds) and subsequent functional changes. The effect of *natural transformation* is defined by Φ(M1) and Φ(M2). Thus, the E(M1) and E(M2) are transformed into the C(M1) and C(M2), which is a function of the *natural transformation,* which is defined by changing the functions from E to C. Thus, the relations of *natural transformation* are denoted as Φ = {Φ(M1): E(M2) ⟶C(M2) | M2$\in$ B}. Then it is namely ‘*commutes’* as follows: Cf o Φ(M1) = Φ(M2) o Ef.

We further explained how on defining topological spaces, including neuronal cells embedded in a growing compact tissue, mathematically. First, this tissue (here, we mean the neuronal tissue) is considered a finite topological space ‘S’, which consists of two elements (X, ${\tau}_m$) that X is the non-countable part, such as the extra-cellular matrix and ${\tau}_m$ is the differentiable *manifold* that is the countable part like the neuronal cells (NC) such that (X $\cap$  ${\tau}_m$)$\ne \varnothing$. Therefore, it satisfies the *Lindelöf space* and is *Hausdorff,* where all topological manifolds belong [43–45]. Thus, there exist subspaces such that U_1_, U_n_  $\subset$ S, (U_1_$\cup$ U_n_) $\subset$ S and (U_1_$\cap$ U_n_) $\epsilon$ S. The ${\tau}_m$ can be defined as differentiable manifolds that are countable and compact. The relation between the NC is as follows if ${NC}_1,\dots, {NC}_i,{NC}_n$ then ${NC}_1\cap{NC}_i\cap{NC}_n\ne \varnothing$. In addition, since the tissue grows continuously and changes in shape from the original tissue structure and morphology, it can be considered as a *quotient space* [18] such that G: (X$,$  ${\tau}_m$) $\to \left(Y,{\tau}_n\right)$. Further, the quotient space could be regarded as a *Banach subspace* where referred to as vector space which is by definition *a Hilbert space* [46]. As we defined the neuronal cells as analogous to a differentiable manifold that is indicated by every point ${p}_{\left\{x,y,z\right\}}$ such that ${R}^n=\left\{\left({x}_1,{x}_i,{x}_n\right):{x}_i\in R\ for\ i=1,n\right\}$and $\left\{\mathrm{x},\mathrm{y},\mathrm{z}\right\}\in{R}^n$ on the differentiable manifold ‘*M’,* which is denoted by $d\left({NC}_{{\boldsymbol{p}}_{\left\{x,y,z\right\}}}\right)$=$\frac{\partial f(M)}{\ {\partial x}_i}$ equation. Next, it is essential to mention the other factors associated with cells independently regulate the fate of cellular development and collectively are reffered to thermodynamic laws [47, 48]. The Gibbs free energy that combines the enthalpy and entropy and thus is related to the thermodynamic laws has a deterministic role if a chemical reaction process can continue in a certain direction [21]. The fundamental equations for Gibbs energy are denoted by $G=U+ pV- TS$, it consists of U, the internal energy; P, the pressure; V, the volume; T, the temperature and S, the entropy. The differential for Gibbs is defined by $dG=- SdT+ Vdp+\sum_{i=1}^N{\mu}_id{n}_i$ in which ${n}_i$ the amount of particles and ${\mu}_i$ the chemical potential of the particle and N, the number of particles [47]. This chemical factor in the form of differential for Gibbs that is described here is assumed to be the contributing factor that leads to the change in the manifolds that are presented in Figure 8 as Functor C: Cf(M1)→Cf(M2).

Further, the electrical activity (E) of the cells is regarded as another independent factor that contributes to the morphological change of the cells (assuming the change in the manifolds shown in Fig. 8 as Functor E: Ef(M1)→Ef(M2)) and leads to cellular growth. The equation is called the Van der Pol equation [7] and is applied to generate the vector field in two-phase, which is ${\dot{x}}_2=-{x}_1-m\left({x}_1^2-1\right){x}_2$, m = 0.1 and m = 1, E$={\dot{x}}_2$.

Finally, we have to define the calculation of Fig. 8D, which is, in summary, the flow of the vector field that affects and interacts with every point. ${p}_{\left\{x,y,z\right\}}$ of the differentiable manifold, $d\left({NC}_{{\boldsymbol{p}}_{\left\{x,y,z\right\}}}\right)$=$\frac{\partial f(M)}{\ {\partial x}_i}.$ It is reminded that there is a unified approach that deals with manifolds with higher dimensions in the field of geometry and topology called differential forms, explained by Cartan [49]. It is applicable when we express the effect of the vector field on the differentiable manifolds. In our model, there are two vector fields E and C, which are denoted by $\alpha{(E)}_k$and$\beta{(C)}_l$on point $p\ \mathrm{such}\ \mathrm{that}\ p\in d\left({NC}_{{\boldsymbol{p}}_{\left\{x,y,z\right\}}}\right)$. There exists (${X}_{S(1)},{X}_{S(i)\dots, }{X}_{S(k)}$)$\in f{(M)}_p$. Thus, their *wedge product* is denoted by the following equation ($\alpha \wedge \beta$)$\big({X}_{S(1)},{X}_{S(i)\dots, }{X}_{S(k)}\big)$=$\frac{1}{K!l!}{\sum}_{S\in S\left(k+1\right)}\operatorname{sign}\left(\mathrm{S}\right)\alpha \big({X}_{S(1)},{X}_{S(i)\dots, }{X}_{S(k)}\big)$*…*  $\beta \big({X}_{S\left(k+1\right)},{X}_{S(i)\dots, }{X}_{S\left(k+1\right)}\big)$. The differential k-form can be defined in general form with ${\alpha}_{i1}\dots{\alpha}_{ik}$= $\alpha \left(\frac{\partial }{\partial{x}^{i1}},\dots, \frac{\partial }{\partial{x}^{ik}}\ \right)$ denoted as a vector field and ${f}_{i1}$ is assigned as coordinate function near point $p\in d\left({NC}_{{\boldsymbol{p}}_{\left\{x,y,z\right\}}}\right)$ and then it yields ${\sum}_{i1<\dots < ik}{\alpha}_{i1}\dots{\alpha}_{ik}d{f}_{i1}\wedge \dots \wedge d{f}_{ik}$) [50]. Furthermore, since statistical equations do not regulate the cellular processes, it requires defining how these interactions between the vector fields and every point on manifolds could be ruled by logical equations such as Boolean algebra at the time ‘*dt’* [51]. Hence, each of the mentioned contributing factors to cellular development $\Big\{{X}_1=\alpha{(E)}_k$, ${X}_2=\beta{(C)}_l,{X}_3=d\left({NC}_{{\boldsymbol{p}}_{\left\{x,y,z\right\}}}\right)\Big\} $ should be included in the *Boolean function* before implementing the above-mentioned equations (Fig. 8B).

Thus, the differential of a Boolean function is defined by $f\left({X}_i\oplus{dX}_i\right)$= $\big({X}_1\oplus{dX}_1,$ … ${X}_i\oplus{dX}_i\big),\mathrm{so}\ \mathrm{the}\ \mathrm{expression}\ \mathrm{of} \left({X}_i\oplus{dX}_i\right)$ implies {if ${X}_i\ value\ chnages\ then\ {dX}_i=1$, otherwise the ${dX}_i=0$}. Therefore, the *total differential function* is defined by ${d}_{X_i}f\left({X}_i\right)$= $f\left({X}_i\right)\oplus f\left({X}_i\oplus{dX}_i\right)$*,* and the *total differential minimum* is denoted by ${\min}_{{\mathrm{X}}_{\mathrm{i}}}f\left({X}_i\right)$= $f\left({X}_i\right)\mathbf{\bigwedge}f\left({X}_i\oplus{dX}_i\right)$ and *total differential maximum* by ${\max}_{{\mathrm{X}}_{\mathrm{i}}}f\left({X}_i\right)$= $f\left({X}_i\right)\boldsymbol{\bigvee}f\left({X}_i\oplus{dX}_i\right)$. The abovementioned equations are summarized and illustrated with an example in Fig. 8.

## Discussion

In this study, we tried to model the neurodevelopmental processes mathematically but, more importantly stressing the possibilities and the pitfalls while categorizing the neurodevelopment for simplicity into various approaches. This process necessitates establishing a platform where these biological phenomena are rigorously defined within the framework of topological spaces, using mathematical terms that closely approximate the objective reality of the underlying dynamics and cellular structure.

We devised three approaches to determine the aspect from which to approach testing the feasibility of neurodevelopmental mathematical modelling. This might not sound as important as mathematical modelling at first glance; however, it is essential to first try to approximate the real dynamics underlying that specific process. Neurodevelopment involves intricate cellular and molecular interactions that are challenging to study solely through experimentation. Our mathematical models may provide a framework to simulate these complex processes, offering insights into the underlying mechanisms governing neurodevelopment.

It does not sound easy because, as we discussed in the Fig. 1, it can be viewed and modelled in all the steps of neurodevelopment instead of just focusing on neuronal clustering during corticogenesis [8]. Comparatively, several theories do not provide a relevant computational formulation for neurodevelopmental processes. Most of the studies provide a mathematical framework for brain growth in the form of networks and wired nodes between different brain regions. They do not offer a specific parametrization for cellular characteristics or how the neurodevelopment process occurs based on mathematical and geometrical parameters similar to what we proposed in this study. Instead, their findings are limited to the nodes and wiring changes and are not established on the underlying mathematical/ geometrical mechanism contributing to neurodevelopment [6]. In another study, they have proposed a probabilistic model for neurodevelopment [52], which is different from what we designed based on the geomatical equation and topological spaces. The probabilistic models does not seem to approximate the reality of neurobiological processes at cellular and subcellular level, since these neurobiological processes look like a robust dynamic system rather than function stochastically.

Furthermore, the complexity of biology requires us to be cautious when we intend to do modelling. Therefore, to address this challenge, we proposed at least two criteria that should precede any considerations of mathematical modeling. It helps to consider them as they are two main elements, so-called naturally built-in, that mathematically should be regarded. They are briefly considered *holomorphic*, indicating continuity, complex-valued function, and group *homomorphism* that can transform into different structures. In the next step, we were interested in *cell division to neurodevelopment*, defined as the orientation and organization of cells influenced by cell division and polarity. As previously mentioned, this pattern and approach naturally occur in cells during division. The actin filaments beneath the cell membrane are the so-called ‘cell cortex’ responsible for cell division and tissue morphogenesis [22, 23]. Consequently, we hypothesized that the *actin filaments* could be used as a basis to mathematically explore how to model the cell division and development. However, further progress in generating the tissue model based on this approach proved challenging. Nevertheless, this is a distinguishable finding when we model biological phenomena mathematically. We should not simply try to push fitting the mathematical equations into the underlying mechanisms existing in biological systems; instead, the critical point is discovering the appropriate approach and approximate mathematical modelling that includes the proper topological definition and providing proper, relevant algorithms for the underlying dynamics or mechanism.

*Next, we tried the neural tube formation to neurodevelopment* approach. The model serves as a formal depiction of growth, where growth emerges from a combination of proliferation, leading to an increase in cell number, and differentiation, which results in a decrease in cell number. It looks fascinating as the neuronal trajectory is projected all along the CNS longitudinally, so the neurons can be modelled accordingly, as we showed in this section and provided a simplified equation that geometrically tries to fit both the structural complexity and space formation.

Finally, our study addressed neural plate formation and neurodevelopment by proposing a comprehensive model that encompasses key elements such as cellular characteristics and tissue growth in the z-axis based on a numerical map. We introduced a proposed equation involving geometrical functions that act as a fitting model, converging into two components through a numerical solution, specifically the *Runge–Kutta method*. [16]. The polynomial equation derived from the binomial formula is intricately linked to the numerical map, notably Pascal’s triangles, and the parameter ‘M’, which governs individual cell growth and development across three axes, thereby regulating tissue development as a whole. This integrated approach provides valuable insights into the complex processes underlying neurodevelopment, offering a framework for further exploration and understanding in this field.

This model meets many of the suggested requirements. However, as we don’t know the exact numerical map, our understanding must be individualized for different tissue types, so we need to adjust the equations further. Further study is required to discover all components and complete this model that can generate the whole brain. However, it was essential to develop a model that can be a platform for mathematical modelling of neurodevelopment. In the last part, we discuss and compare our proposed equation with the closest equivalent to our equation, the *Weierstrass Elliptic Function*. This equation uses lattice and *p-function* to generate the 3D objects in periodicity. This model has difficulty providing the proper parameters that can be adjusted for, e.g., the cell characteristics, controlling the continuous tissue growth (brain development) in z axis, or even mapping the growth. Therefore, in our experience, it would be almost impossible to provide a model with this equation without considering these issues. In final section of the results describes mathematical concepts that reflect the process of neurodevelopment in terms of topological definition and changes in the manifolds interacting with the vector fields in the form of electrical and cellular Gibbs energy that all contribute to cell growth and tissue formation. A well-known diagram of Functors, Exterior Algebra, and Boolean functions was employed to illustrate and elucidate the process in mathematical terms and topological spaces. This information is helpful to clarify the details of neurodevelopment from topological perspective and enhances understanding neurodevelopment in terms of mathematical terminology.

In summary, our mathematical model incorporates cellular characteristics, such as cell division, organization, and polarity, to simulate the behavior of cells during neurodevelopment. Additionally, the model includes equations and algorithms that describe tissue growth in three dimensions, reflecting the spatial organization and development of neural tissues during embryogenesis.

### Limitation

The study acknowledges the need for further refinement of the proposed equations and models. Adjustments may be required to account for different tissue types and to ensure that the models accurately represent the dynamics of neurodevelopment across various stages. Moreover, our models may face challenges in providing proper parameters that can be adjusted for factors such as cell characteristics and tissue growth in the z-axis.

## Conclusion

For the first time, we defined how to mathematically approach neurodevelopment regarding the underlying dynamics of neurodevelopment from different main aspects. We provided a unique model with details and a novel equation expected to fully model the whole brain tissue if the numerical map is discovered. Finally, we could define the process of neurodevelopment through a topological definition that includes the interaction of the manifolds with the vector fields in the form of electrical and cellular Gibbs energy. Finally, our study integrates biological insights with mathematical formulations, aiming to bridge the gap between biological understanding and mathematical modeling of neurodevelopmental processes. By proposing novel equations and algorithms, the study provides a platform for researchers in the field of artificial neural networks and quantum computation to potentially develop models that autonomously grow and develop with electrochemical properties similar to the real brain. That may include better diagnosis of diseases or advancements in healthcare technologies. To enhance this study, further study could focus on integrating topological definitions more effectively with a detailed characterization of cellular components, including the cell cytoskeleton and ion channels, encompassing both their electrical and chemical properties. By achieving a more comprehensive representation of neuronal structure and function, coupled with an appropriate numerical mapping, the resulting model would better approximate the intricate reality of neuronal development within mathematical and geometrical frameworks. Thus, by applying these modellings, there is a chance to generate an artificial neural network that autonomously grows and develops with electrochemical properties similar to real brain. Hence, in case of improvement in artificial neural network, there is a chance to be used for health care system benefit such as improvement in diagnosis of neurological disorders.

**Key Points**

- Using mathematical models help understand the complex processes involved in embryonic neurodevelopment.
- By combining biological knowledge with mathematical modeling, researchers can gain insights into the processes of neural cell differentiation and organization.
- Equations and algorithms that describe tissue growth in three dimensions, reflecting the spatial organization and development of neural tissues during embryogenesis.
- Actin filaments in mathematical modeling is important to understand cell division and tissue morphogenesis.

## Data Availability

All data reported in this paper will be shared by the lead contact upon request.
